# Dataset on the analysis of β-galactosidase immobilization efficiency on aminomethyl polystyrene (AMP) resin in syringe and column reactors

**DOI:** 10.1016/j.dib.2025.112389

**Published:** 2025-12-15

**Authors:** Tabea Boehme, Bernadette Straub, Ursula Eschenhagen, Magnus S. Schmidt

**Affiliations:** Institute of Precision Medicine, Organic and Bioorganic Chemistry Labs, Faculty Health Medical and Life Sciences, Furtwangen University, Jakob-Kienzle-Str. 17, D-78054 VS-Schwenningen, Germany

**Keywords:** Covalent enzyme immobilization, Β-galactosidase, ONPG, Lactose

## Abstract

β-Galactosidase was covalently immobilized on AMP resin using a p-phenylenediisothiocyanate (PDC) linker. Resin loading was determined by measuring the protein concentration difference between the original lactase solution and the reactor supernatant using a Bradford assay. Enzyme activity of the supernatants was assessed with ONPG as the substrate, and lactose turnover rates were measured using a lactose solution. The dataset provides detailed results for five different approaches with several syringe reactors and two column reactors, enabling comparison of immobilization efficiency and catalytic performance. The dataset may be valuable for industrial applications where stable and efficient immobilized β-galactosidase systems are required, such as lactose-free dairy production.

Specifications Table

**Table:** 

Subject	Engineering & Materials science
Specific subject area	Covalently immobilized β-Galactosidase on AMP resin using a PDC linker.
Type of data	Table, Analyzed, Figure
Data collection	The data were collected by conducting several analytical experiments to evaluate the success of the immobilization. A Bradford assay was performed with the original lactase solution and the reactor supernatant after immobilization to calculate the amount of immobilized lactase. In addition, an enzyme kinetics assay was carried out for the original lactase solution and the reactor supernatant, using ONPG as the substrate. The values from the linear phase of the reaction were used to calculate enzyme activity. Finally, the lactose turnover rate in the reactors was determined by filling them with a 4.7 g/L lactose solution and measuring lactose conversion after 6.5 h.
Data source location	Institute of Precision Medicine Organic and Bioorganic Chemistry Labs Faculty Health, Medical and Life Sciences Hochschule Furtwangen | Furtwangen UniversityJakob-Kienzle-Str. 17, 78054 Villingen-Schwenningen Germany
Data accessibility	1. With the article: [1] 2. Repository name: Mendeley Data Data identification number: DOI: 10.17632/pxvtgd82dn.1 Direct URL to data: https://data.mendeley.com/datasets/pxvtgd82dn/1
Related research article	Boehme T, Straub B, Eschenhagen U, Schmidt M. Next Level of p-Phenylene Diisothiocyanate-based Covalent Immobilization of β-d-Galactosidase: technical optimization an application. Journal of Biotechnology. 2025; submitted [2].

## 1 Value of the Data

• This dataset might be valuable for the industry for establishing a more sustainable way of producing lactose-free products.
• Provision of comprehensive data on the analysis of immobilization efficiency using a PDC linker to immobilize the β-galactosidase covalently on AMP resin. This includes resin loading, enzyme activity and the turnover rate of lactose after 6.5 h.
• Comparison between the immobilization success of two different reactor types, the syringe reactor and the column reactor.

## 2 Background

Due to its widespread prevalence in the global population, lactose intolerance holds a central relevance in nutrition and the food industry. Lactose-free dairy products can be produced by enzymatic hydrolysis of lactose using β-Galactosidase [3,4]. Currently, industrial processes rely on soluble enzymes added directly to the product, which restricts their use to a single process cycle [5]. This limitation can be overcome by immobilizing lactase covalently to resin beads. An initial study on covalently immobilizing lactase using p-phenylenediisothiocyanate has already been published and forms the basis of the presented dataset [6].

This dataset was compiled to analyze the efficiency of the immobilized lactase in two different reactor types. Initially, lactase was immobilized in syringe reactors. To examine the feasibility of large-scale economic processes, an upscale in immobilization from the syringe reactors to a column reactor was therefore carried out.

## 3 Data Description

This section describes the data of the analysis of the immobilization efficiency as well as the data collected to assess the stability of the lactase in the column reactor over several days.

### 3.1 Analysis of the immobilization efficiency

Table 1 shows a summary of the data obtained from the immobilization experiments. The table is structured according to four main experimental approaches (Approach 1 – 4) done on different days, each including a varied number of reactors. Additionally, data from a previous work are included for comparison, denoted as Approach Straub, marking the first approach including a column as a reactor instead of syringe reactors. Approach 1 also uses a column as a reactor while the other approaches (Approach 2 – 4) are done in a syringe reactor.

**Table 1:** Data overview for the analysis of the immobilization.

Approach	Reactor	Resin loading (mg/g)	Enzyme activity (mkat)	Maximum lactose turnover in the reactor
Approach 1	Column	0.401	0.252	Not applicable
Approach 2	R1	0.619	0.211	51.60 %
	R2	1.18	0.191	57.60 %
	R3	1.13	0.217	61.20 %
	R4	1.223	0.177	30.80 %
Approach 3	R1	2.336	0.170	55.70 %
	R2	1.555	0.170	54.20 %
	R3	2.555	0.134	43.30 %
	R4	Same as above	Same as above	51.50 %
	R5	2.222	0.181	49.10 %
	R6	2.132	0.160	52.30 %
	R7	1.791	0.174	50.00 %
	R8	1.555	0.178	45.60 %
	R9	3.068	0.113	49.90 %
	R10	3.881	0.077	57.80 %
Approach 4	R1	1.083	0.159	87.80 %
	R2	5.135	0.032	39.50 %
	R3	2.44	0.154	83.40 %
	R4	5.511	0.004	35.70 %
	R5	1.189	0.164	82.40 %
	R6	2.145	0.179	86.30 %
	R7	2.42	0.167	98.80 %
	R8	1.86	0.153	91.80 %
	R9	1.911	0.161	92.60 %
	R10	1.891	0.175	96.90 %
Approach Straub	R1	2.113	0.000	Not applicable
	R2	1.946	−0.006	
	R3	2.181	0.000	
	R4	1.906	0.006	
	R5	2.062	0.000	
	R6	1.765	0.006	
	R7	2.038	0.000	
	R8	1.768	0.000	
	R9	1.933	0.000	
	R10	2.082	0.000	
	R11	1.911	0.000	
	R12	2.111	0.001	
	R13	1.886	0.006	
	R14	1.913	0.001	
	Column	0.627	0.022	

In approach 3, the reactor supernatant of reactors 3 and 4 was accidentally put into the same container, leading to the combined analysis of the reactors. Since the turnover rate was analyzed directly in the reactor, these results could have been analyzed separately for each reactor.

A tendency for higher resin loading in the syringe reactor can be seen in the syringe reactors compared to the two-column reactors. Conversely, the enzyme activity seems higher in the column reactors compared to the syringe reactors. The turnover rate of lactose in the reactor was only recorded in syringe reactors excluding, the Straub approach. Turnover rates vary within the reactors showing yields between 35 % and 98 %, while the majority of reactors show a yield of around 50 %.

### 3.2 Stability study over several days in the column

**Fig. 1:**
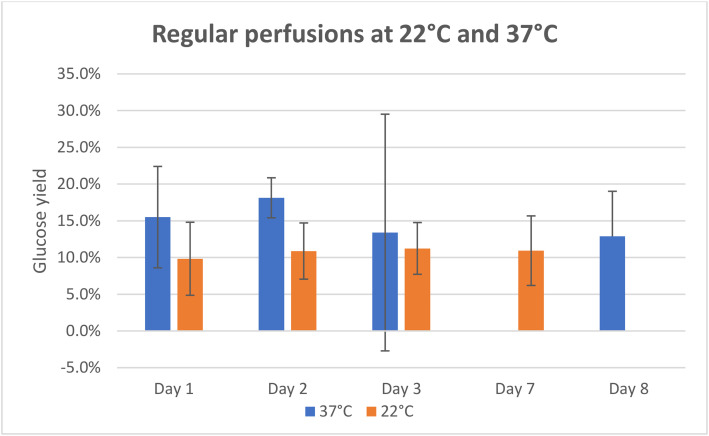
Glucose yields of perfusions on different days to examine the stability of the lactase.

In Fig. 1, the average glucose yields of the regular perfusions can be seen. The experiments were conducted over three consecutive days, followed by an additional perfusion several days later. The final perfusion at 22 °C was carried out after 7 days, while the one at 37 °C was conducted after 8 days due to variations in holiday length. At 22 °C, the glucose yield remained consistent at approximately 10 % across all days. In contrast, the yields at 37 °C were around 15 %, with only minor fluctuations.

## 4 Experimental Design, Materials and Methods

### 4.1 Experimental design

In Table 2, all devices used during the experiments are shown, assigned with their model name and manufacturer. All chemicals and Kits used are listed in Table 3 with their structural formula or abbreviation, the manufacturer and either their CAS-number or article number. The immobilization took place in two different kinds of reactors. One was a 20 mL syringe reactor (Carl Roth GmbH + Co. KG, Karlsruhe, Germany) and the other was a 40 mL glass column with an integrated tempering jacket (TH.Geyer GmbH & Co. KG, Renningen, Germany).

**Table 2:** List of used devices.

Device	Model name	Manufacturer
Incubator	Certomat® H	B.Braun Melsungen AG, Melsungen, Germany
Incubator	TH 15	Edmund Bühler GmbH, Bodelshausen, Germany
Magnetic stirrer	PCE-MSR 100	PCE Deutschland GmbH, Meschede, Germany
Overhead shaker	Reax 2	Heidolph Instruments GmbH & Co. KG, Schwabach, Germany
pH-Meter	-	Mettler Toledo
Pump	Reglo	Ismatec® – Cole-Parmer GmbH, Wertheim, Germany
Thermostatic bath	Thermomix ME 852 112/3	B.Braun Melsungen AG, Melsungen, Germany
UV–Vis spectrophotometer	UVmini-1240	Shimadzu Corporation, Kyoto, Japan
Water bath	WBT 12	meding Lab GmbH, Freital, Germany

**Table 3:** List of the used chemicals and kits.

Chemical/Kit	Structural formula/Abbreviation	Manufacturer	CAS-number/Article number
3-(N-Morpholino)propanesulfonic acid	MOPS	Carl Roth GmbH + Co. KG, Karlsruhe, Germany	1132-61-2
Ammonium sulfate	H_8_N_2_O_4_S	Carl Roth GmbH + Co. KG, Karlsruhe, Germany	7783-20-2
Bovine serum albumin	BSA	Carl Roth GmbH + Co. KG, Karlsruhe, Germany	9048-46-8
Protein Assay Dye Reagent Concentrate	Bradford assay	Bio-Rad Laboratories, Inc., Hercules, CA, USA	7664-38-2
Calcium chloride dihydrate	CaCl₂ x 2H₂O	Riedel-de Haën AG, Seelze, Germany	10035-04-8
Diisopropylethylamine	DIPEA	AAcros Organics, Geel, Belgium	7087-68-5
Dimethylformamide	DMF	NeoFroxx GmbH	68-12-2
Dimethyl sulfoxide	DMSO	Carl Roth GmbH + Co. KG, Karlsruhe, Germany	67-68-5
Disodium hydrogen phosphate dihydrate	Na₂HPO₄ x 2H₂O	Merck KGaA, Darmstadt, Germany	10028-24-7
Enzytec™ Liquid d-Glucose	Glucose Test Kit	R-Biopharm AG, Darmstadt, Germany	E8140
Ethylenediaminetetraacetic acid disodium salt dihydrate	EDTA−Na₂ × 2 H₂O	Carl Roth GmbH + Co. KG, Karlsruhe, Germany	6381-92-6
Lactase 13.000 EXPRESS	Lactase tablet	sanotact GmbH, Münster, Germany	-
Lactose monohydrate	C₁₂H₂₂O₁₁ x H₂O	Carl Roth GmbH + Co. KG, Karlsruhe, Germany	10039-26-6
Magnesium chloride heptahydrate	MgCl₂ x 7H₂O	Prepared in the lab	-
Magnesium sulfate heptahydrate	MgSO₄ x 7H₂O	Merck KGaA, Darmstadt, Germany	10034-99-8
Manganese(II) chloride stock solution (20 g/L)	MnCl₂ stock solution Prepared in the lab	Prepared in the lab	-
Sodium bicarbonate	NaHCO₃	AppliChem GmbH, Darmstadt, Germany	144-55-8
Sodium carbonate	Na₂CO₃	Carl Roth GmbH + Co. KG, Karlsruhe, Germany	497-19-8
Sodium chloride	NaCl	Häberle LABORTECHNIK GmbH & Co. KG	7647-14-5
Sodium dihydrogen phosphate dihydrate	NaH₂PO₄ x 2H₂O	Carl Roth GmbH + Co. KG, Karlsruhe, Germany	13472-35-0
o-Nitrophenyl-β-d-galactopyranoside	ONPG	Carl Roth GmbH + Co. KG, Karlsruhe, Germany	369-07-3
Polysorbat 20	Tween®20	Sigma-Aldrich, St. Louis, MO, USA	9005-64-5
Polystyrol AN NH₂	AMP-Harz	Rapp Polymere GmbH, Tübingen, Germany	H50056002.25G
Potassium dihydrogen phosphate	KH₂PO₄	Carl Roth GmbH + Co. KG, Karlsruhe, Germany	7778-77-0
p-Phenylenediisothiocyanate	PDC	Sigma-Aldrich, St. Louis, MO, USA	4044-65-9
Propan-2-ol	C₃H₈O	Carl Roth GmbH + Co. KG, Karlsruhe, Germany	67-63-0
Protein Assay Dye Reagent Concentrate	Bradford solution	Bio-Rad Laboratories,Inc., Hercules, CA, USA	7664-38-2

### 4.2 Preparation of solutions

#### 4.2.1 Preparation of phosphate buffer saline (PBS buffer): 200 mM, pH 6.5

Potassium dihydrogen phosphate (2.72 g), ammonium sulfate (0.88 g) and magnesium sulfate heptahydrate (0.04 g) were dissolved in deionized water. The pH was adjusted to 6.5 using potassium hydroxide, and the solution was made up to a final volume of 100 mL with deionized water.

#### 4.2.2 Preparation of phosphate buffer saline (PBS buffer): 200 mM, pH 7.3

Sodium dihydrogen phosphate dihydrate (0.365 g), disodium hydrogen phosphate dihydrate (1.37 g), sodium chloride (8.75 g), magnesium chloride heptahydrate (0.02 g) and calcium chloride dihydrate (0.013 g) were dissolved in deionized water. 1 mL of manganese (II) chloride stock solution (159.0 mM) was added and the pH was adjusted to 7.3 using 0.1 M hydrogen chloride. The prepared solution was filled up to 1 liter.

#### 4.2.3 Preparation of phosphate buffer saline with tween® 20 and 20 % (v/v) DMSO (PBS-T 20 % (v/v) DMSO)

400 µL of Tween® 20 was added to 800 mL of PBS buffer (pH 7.3) and diluted with 200 mL of DMSO.

#### 4.2.4 Preparation of carbonate buffer: 20 mM, pH 10.1

Sodium carbonate (1.064 g) and sodium bicarbonate (0.84 g) were dissolved in deionized water. The pH was adjusted to 10.1 using sodium hydroxide, and the solution was filled up to 1 L with deionized water.

#### 4.2.5 Preparation of PEM buffer: 199.9 mM phosphate, pH 6.5

Potassium dihydrogen phosphate (27.2 g), disodium ethylenediaminetetraacetate dihydrate (0.034 g), and manganese (II) chloride heptahydrate (0.02 g, 0.06 mM) were dissolved in deionized water. The pH was adjusted to 6.5 using potassium hydroxide, and the solution was filled up to 1 L

#### 4.2.6 Preparation of PDC linker solution: 260.0 mM

p-Phenylenediisothiocyanate (0.5 g) was dissolved overnight in 5 mL of dimethyl sulfoxide (DMSO) using a magnetic stirrer. Then, 40 µL of N,N-diisopropylethylamine (DIPEA) was added and the solution was filled up to 10 mL with DMSO.

#### 4.2.7 Preparation of lactase stock solution: 260 FCC/mL

One lactase tablet was dissolved in 50 mL of phosphate-buffered saline (PBS, pH 7.3).

#### 4.2.8 Preparation of sodium carbonate solution (100 g/L): 943.2 mM

10 g of sodium carbonate was dissolved in 100 mL of deionized water.

#### 4.2.9 Preparation of ONPG stock solution: 10 mM

0.301 g of o-nitrophenyl-β-d-galactopyranoside (ONPG) was dissolved in 100 mL of PEM buffer. All containers were protected from light using aluminum foil.

#### 4.2.10 Preparation of 0.5 mM ONPG working solution

5 mL of ONPG stock solution was diluted with 95 mL of PEM buffer. All containers were protected from light using aluminum foil.

#### 4.2.11 Preparation of BSA stock solution (1 mg/mL): 15.0 µM

0.5 g of bovine serum albumin (BSA) was dissolved in 50 mL of PBS buffer (pH 6.5).

#### 4.2.12 Preparation of Bradford working solution (1:5 dilution)

20 mL of Bradford reagent was diluted with 80 mL of deionized water.

#### 4.2.13 Preparation of lactose solution (4.7 g/L): 13.0 mM

2.093 g of 3-(N-morpholino)propanesulfonic acid (MOPS) was dissolved in 1 L of deionized water and 4.95 g of lactose monohydrate was added to the 1 L of MOPS buffer.

### 4.3 Immobilization of lactase on aminomethyl polystyrene resin

The immobilization was performed in different reactor types (column, 10 mL Syringe, 20 mL Syringe). Due to the varying reactor volumes, the amounts of immobilized lactase and reagents differed accordingly (Table 4).

**Table 4:** Amounts of reagents corresponding to the reactor type.

Reactor	AMP resin	DMF	PDC linker solution	Wash step 1	Lactase solution	Wash step 2
Column	4.0 g	40 mL	16 mL	2 × 40 mL isopropanol 2 × 40 mL deionized water 2 × 40 mL carbonate/ bicarbonate buffer 1 × 40 mL deionized water	16 mL	4 × 40 mL PBS-T 20 % (v/v) DMSO
Syringe 10 mL	0.5 g	5 mL	2 mL	2 × 5 mL isopropanol 2 × 5 mL deionized water 2 × 5 mL carbonate/ bicarbonate buffer 1 × 5 mL deionized water	2 mL	4 × 5 mL PBS-T 20 % (v/v) DMSO
Syringe 20 mL	1.0 g	10 mL	4 mL	2 × 10 mL isopropanol 2 × 10 mL deionized water 2 × 10 mL carbonate/ bicarbonate buffer 1 × 10 mL deionized water	4 mL	4 × 10 mL PBS-T 20 % (v/v) DMSO

First, aminomethyl polystyrene resin was swollen in DMF for 24 h at 6 °C. After removing excess DMF, PDC linker solution was added and incubated for 3 h, followed by sequential washing: twice with 2-propanol, twice with deionized water, twice with carbonate/bicarbonate buffer, and once with deionized water. Next, lactase solution was added and incubated for 24 h at room temperature. During this step, syringes were rotated on an overhead shaker at the lowest speed, while the column was shaken at 50 rpm on a platform shaker and padded. After draining, the reactor was washed four times with PBS-T containing 20 % (v/v) DMSO. To prevent enzyme desiccation, a small volume of this solution was left inside. Reactors were sealed and stored at 6 °C.

### 4.4 Bradford test

The protein content of the immobilized enzyme was determined using a Bradford assay following the manufacturer’s protocol. Measurements were performed in duplicate. A calibration curve was generated using a dilution series from 0 mg/mL BSA to 0.9 mg/mL BSA in 0.1 mg/mL steps using a BSA stock solution (1mg/mL) in PBS buffer (pH 6.5).

For the assay, 100 µL of each dilution and sample (reactor supernatant and original lactase solution) were added to 5 mL of Bradford solution 1:5 dilution and incubated for a minimum of 5 min and a maximum of 1 hour. Measurements were taken at 595 nm using a UV–VIS spectrophotometer. Protein concentration was calculated using the linear regression equation of the standard curve. The amount of immobilized enzyme was determined by subtracting the protein concentration in the reactor supernatant from the amount of the initial lactase solution (see Eq. (1))

(1) $$C_{Protein,\;immobilized}=\;C_{Protein,\;initial\;Lactse\;solution}-\;C_{Protein,\;Reactor\;supernatant}$$

Based on the calculated protein concentration of immobilized lactase in the reactor, the resin loading was determined using Eq. (2).

(2) $$\zeta =\;\frac{C_{Protein,\;immobilized}*\;V_{Lactase}}{m_{resin}}$$

### 4.5 Enzyme activity

Enzyme activity was determined from time-resolved kinetic measurements using an ONPG solution (0.5 mM) as substrate. Samples were diluted 1:20 with PBS buffer (pH 6.5) and incubated at 37 °C in a water bath together with the ONPG solution. For each assay, 5 mL of enzyme solution was mixed with 15 mL of ONPG solution and the reaction was recorded over 60 seconds by taking 1 mL samples every 10 s from the start of the reaction. Each sample was immediately mixed with sodium carbonate to stop the reaction and afterwards measured spectrophotometrically at a wavelength of 420 nm.

To correct the measured values, the solutions listed in Table 5 were also determined and the correction was applied using Eq. (3).

(3) $$\Delta A_{corrected}\;=\;\left(A_{sample}\;-\;A_{ONPGwithoutEnzyme}\right)-\;\left(A_{BufferwithEnzyme}\;-\;A_{BufferwithoutEnzyme}\right)$$

(4) $$c=\frac{A}{\varepsilon *d}$$

**Table 5:** Solutions needed to determine the correction values.

	Sodium carbonate	PBS buffer (pH 6.5)	ONPG solution	Sample solution	PEM buffer
ONPG without Enzyme	0.5	0.25	0.75		
Buffer with Enzyme	0.5	0.75		0.25	
Buffer without Enzyme	0.5	0.75			0.25

The concentration was calculated in two ways, one using the Lambert Beer’s law [7] (Eq. (4)) and the other using a calibration curve done by Matteo Haese (Eq. (5)).

c: Concentration of ONP (mol/l)
A: Absorption at 420 nm
ε: Extinction coefficient at 420 nm (4500 mol−1 · cm−1)
d: Thickness of the cuvette (= 1 cm)
  (5) y=4.3124x+0.031

The linear increase in product concentration between 20 and 40 s was used to calculate the enzyme activity. The full calculation can be seen in Eq. (6).

(6) $$Enzyme\;activity=\frac{\Delta C_{ONP}*V_{Cuvette}}{\Delta t*V_{Enzyme}}$$

### 4.6 Lactose turnover rate in the reactor

The glucose turnover rate of a 4.7 g/l lactose solution was determined over a period of 6.5 h in the syringe reactor. The reactor was filled with lactose solution at maximum and incubated in the overhead shaker at the lowest speed and 37 °C under the incubation hood. Every 30 min a 0.5 mL sample was taken and stored on ice or at −19 °C until evaluation with a d-glucose test kit, following the manufacturer’s instructions, with the exception that all volumes were reduced by half, to calculate the glucose concentration. The glucose yield was calculated as shown in Eq. (7).

(7) $$Glucose\;yield\;\left(\%\right)=\frac{C_{max.,\;actual}}{C_{max.,\;theoretical}}*100\;\%$$

### 4.7 Column perfusion with a 4,7 g/l lactose solution for stability assessment of the immobilized lactase over time

The stability of the immobilized lactase solution was examined by a column perfusion of a 4,7 g/l lactose solution on four different days with the longest gap between examinations of four days. The lactose solution was passed through the column using a pressure system operating at 0.2 to 0.4 bar. A pump was used to transport the solution through the column at a specific speed. On each day, the lactose solution sample coming out of the column was collected for ten minutes for a total of 60 min. All samples were evaluated using a d-glucose test kit, following the manufacturer’s instructions, with the exception that all volumes were reduced by half. The yields were again calculated as shown in Eq. (7).

## Limitations

A limitation of the dataset is the potential inaccuracy of the glucose quantification using the d-Glucose test kit. Variations in the sensitivity may have caused minor deviations creating several outliers. While all samples were processed under identical conditions to ensure comparability, absolute glucose concentrations should be interpreted with caution.

## Ethics Statement

No experiments on human participants or animals were carried out during the implementation of these experiments, nor were any human or animal rights violated. Additionally, no data was collected from social media platforms.

## CRediT authorship contribution statement

**Tabea Boehme:** Writing – original draft, Data curation, Investigation. **Bernadette Straub:** Data curation, Investigation. **Ursula Eschenhagen:** Resources, Methodology. **Magnus S. Schmidt:** Conceptualization, Supervision, Writing – review & editing.

## Data Availability

Mendeley DataDataset on the analysis of β-galactosidase immobilization efficiency on AMP resin in syringe and column reactors (Original data).

## Appendix Supplementary materials

Image, application 1
