# Supplementary material for: Dataset on the analysis of β-galactosidase immobilization efficiency on aminomethyl polystyrene (AMP) resin in syringe and column reactors
Source: Data Brief. 2025 Dec 15;64:112389. doi: 10.1016/j.dib.2025.112389 (PMC12813484; doi:10.1016/j.dib.2025.112389)
Supplement: Supplementary file 1 [file mmc1.pdf]

Journal of Biotechnology

# Next Level of p-Phenylene Diisothiocyanate-based Covalent Immobilization of β-d-Galactosidase: technical optimization an application

--Manuscript Draft--

| | |
|---|---|
| Manuscript Number: | JBIOTEC-D-25-01145 |
| Article Type: | Research Paper |
| Section/Category: | Biochemical Engineering/Bioprocess Engineering |
| Keywords: | covalent enzyme immobilization;  fixed-bed reactor;  β-D-galactosidase;  lactose hydrolysis;  Aminomethylated polystyrene resin;  phenylenediisothiocyanate |
| Corresponding Author: | Magnus S Schmidt<br>Furtwangen University Institute of Precision Medicine<br>GERMANY |
| First Author: | Tabea L Boehme |
| Order of Authors: | Tabea L Boehme |
| | Bernadette Straub |
| | Ursula Eschenhagen |
| | Magnus S Schmidt |
| Abstract: | In this study, a continuous lactose hydrolysis process in a fixed-bed reactor was developed using β-galactosidase covalently immobilized on resin beads via 1,4-phenylenediisothiocyanate (PDC) as linker. Process conditions, including temperature, enzyme loading, perfusion speed, and repeated perfusion of the same substrate solution were systematically varied. The highest glucose yields were obtained at 55 °C, with increased yields observed at low perfusion speeds, high enzyme loadings, and upon repeated perfusions. Under optimized cycle perfusion over 72 h, final lactose conversion reached approximately 90% at 37 °C and 80% at 22 °C. A hydrolysis process in a fixed-bed reactor was successfully established, although further optimization is required. |

Powered by Editorial Manager® and ProduXion Manager® from Aries Systems Corporation

Furtwangen University · Jakob-Kienzle-Str. 17 · 78054 Villingen-Schwenningen · Germany

Prof. Dr. Magnus Schmidt
Faculty Health, Medical and Life Sciences
Organic and Bioorganic Chemistry Labs
Institute of Precision Medicine
Tel. +49.7720.307-4613
magnus.schmidt@hs-furtwangen.de
10.09.2025

## Cover Letter

Dear Editor-in-Chief,

Please find enclosed our manuscript, “Next Level of p-Phenylene Diisothiocyanate-based Covalent Immobilization of β-D-Galactosidase: technical optimization an application ” which we would like to submit for publication as a article within “Journal of Biotechnology” .

In this article we present the development and technical optimization of a fixed-bed reactor using immobilization techniques of galactosidase on amino functionalizes polystyrene resins for the purpose of technical lactose hydrolysis. The results are very promising indicating good possibilities for technical application. Therefore, we believe our findings would appeal to the readership of the Journal of Biotechnology.

We confirm that this manuscript has not been published elsewhere and is not under consideration by another journal. All authors have approved the manuscript and agree with its submission to the Journal of Biotechnology. We also added a co-submission to Data in Brief including the research data and methods as well as protocols related to this manuscript.

Please address all correspondence to magnus.schmidt@hs-furtwangen.de

We look forward to hearing from you at your earliest convenience.

With my best regards, sincerely yours,

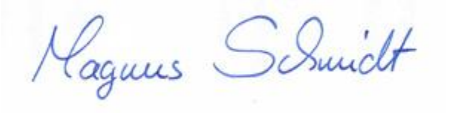

Prof. Dr. Magnus Schmidt

**FURTWANGEN UNIVERSITY**

FURTWANGEN
Robert-Gerwig-Platz 1
78120 Furtwangen
Germany
Tel. +49.7723.920-0
Fax +49.7723.920-1109

VILLINGEN-SCHWENNINGEN
Jakob-Kienzle-Straße 17
78054 Villingen-Schwenningen
Germany
Tel. +49.7720.307-0
Fax +49.7720.307-3109

TUTTLINGEN
Kronenstraße 16
78532 Tuttlingen
Germany
Tel. +49.7461.1502-0
Fax +49.7461.1502-6201

info@hs-furtwangen.de
www.hs-furtwangen.de

**HIGHLIGHTS:**

- A fixed-bed reactor was developed for lactose hydrolysis purposes.
- Therefore, β-galactosidase has been covalently immobilized.
- Process parameters have been optimized including perfusion speeds and temperature.
- Long term experiments showed very good results.

Manuscript Click here to view linked References

# Next Level of p-Phenylene Diisothiocyanate-based Covalent Immobilization of β-D-Galactosidase: technical optimization an application

*Tabea L. Boehme, [a] Bernadette Straub [a], Ursula Eschenhagen, [a,b] Magnus S. Schmidt*[a,c]*

[a] Faculty Health Medical and Life Sciences, Furtwangen University, Campus Villingen-Schwenningen, Jacob-Kienzle-Str. 17, 78054 Villingen-Schwenningen, Germany

Email: boehmetabea@gmail.com

Email: bernadette@straub.id

[b] Institute of Applied Biology, University Pilot Plant/Technical Center, Furtwangen University

Email: ursula.eschenhagen@hs-furtwangen.de

[c] Corresponding author, Institute of Precision Medicine, Organic and Bioorganic Chemistry Lab, Furtwangen University

E-Mail: magnus.schmidt@hs-furtwangen.de

**KEYWORDS:** covalent enzyme immobilization, fixed-bed reactor, β-D-galactosidase, hydrolysis, lactose, Aminomethylated polystyrene resin, phenylenediisothiocyanate

**ABSTRACT:** In this study, a continuous lactose hydrolysis process in a fixed-bed reactor was developed using β-galactosidase covalently immobilized on resin beads via 1,4-phenylenediisothiocyanate (PDC) as linker. Process conditions, including temperature, enzyme loading, perfusion speed, and repeated perfusion of the same substrate solution were systematically varied. The highest glucose yields were obtained at 55 °C, with increased yields observed at low perfusion speeds, high enzyme loadings, and upon repeated perfusions. Under optimized cycle perfusion over 72 h, final lactose conversion reached approximately 90 % at 37 °C and 80 % at 22 °C. A hydrolysis process in a fixed-bed reactor was successfully established, although further optimization is required.

## 1 INTRODUCTION

Due to its wide spread prevalence in the global population, lactose intolerance gains a central importance in nutrition, rising interest in production of lactose free alternatives for the food industry. An economically efficient way of producing lactose free dairy products uses the hydrolysis properties of the enzyme β-Galactosidase (1, 2). Currently, all industrially applicated processes for Lactase free dairy products use soluble Lactase that is added to the product (3). Hence, the Lactase enzyme can only be used for a single process, leading to higher production costs (4). This leads to higher market costs of the product, lowering the acceptance on the customer side (5). Immobilizing Lactase covalently can bypass that limitation, reducing production costs and create a more sustainable way of producing lactose free nutrition (6). In addition, immobilizing enzymes offers further advantages, such as a higher operational stability, as well as the absence in the final product leading to a lower risk of Maillard reactions (7, 5). An initial study on covalently immobilized Lactase using p-phenylenediisothiocyanate as linker was already published and builds the basis of this paper (8). Unfortunately, immobilizing β-Galactosidase is also accompanied by several disadvantages including risk of microbial contamination and lower enzyme activity (9). The latter expresses the need of technical improvement, to achieve a complete conversion of lactose. In this study, a continuous hydrolysis process was established and optimized. For this, different parameters, such as temperature, outflow speed, number of perfusion cycles and amount of Lactase were examined, to establish improved process conditions. The resulting conditions were then implemented in a cycle perfusion, lasting several days, to examine the best possible degree of hydrolysis under these conditions.

## 2 MATERIALS AND METHODS

The following section will give an overview of the used materials and methods to implement and optimize hydrolysis of lactose by β-Galactosidase.

### 2.1 Materials

β-Galactosidase (from A. oryzae (10)) was purchased from sanotact GmbH (Münster, Germany) in the form of Lactase tablets. Lactose monohydrate was purchased from Carl Roth GmbH & Co. KG (Karlsruhe, Germany). Enzytec™ Liquid D-Glucose Test-Kits were obtained from R-Biopharm AG (Darmstadt, Germany). All solvents and other chemicals were of analytical grade. Two glass columns, one with a heating jacket and one without, were purchased from Th. Geyer GmbH & Co. KG (Renningen, Germany).

### 2.2 Solutions and buffer for the column perfusion experiments

**Preparation of phosphate buffer saline (PBS buffer):** 11.8 mM, pH 7.3

Sodium dihydrogen phosphate dihydrate (0.365 g), disodium hydrogen phosphate dihydrate (1.37 g), sodium chloride (8.75 g), magnesium chloride heptahydrate (0.02 g) and calcium chloride dihydrate (0.013 g) were dissolved in deionized water. 1 ml of manganese (II) chloride stock solution (159.0 mM) was added and the pH was adjusted to 7.3 using 0.1 M hydrogen chloride. The prepared solution was filled up to 1 liter.

**Preparation of phosphate buffer saline with Tween® 20 and 20% (v/v) DSMO (PBS-T 20% (v/v) DMSO):** 11,8 mM, pH 7,3

400 µl of Tween® 20, 800 ml of PBS buffer (pH 7.3) and 200 ml of DMSO were mixed.

**Preparation of lactose solution (4.7 g/L)**: 13.0 mM

2.093 g of 3-(N-morpholino)propanesulfonic acid (MOPS) was dissolved in 1 liter of deionized water and 4.95 g of lactose monohydrate was added to the 1 liter of MOPS buffer.

## 2.3 Methods

Immobilized Lactase was inserted into a glass column, to build a fixed bed reactor. Different experiments were conducted to optimize the degradation degree of lactose during perfusion through the reactor.

**Immobilization of β-Galactosidase**

The immobilization procedure and efficiency data of the Lactase used in this study have been published previously (Boehme et al., (11)). The approaches and reactors were screened before their selection for usage. An overview of the used immobilized reactor for each experiment can be seen in Table 1: Perfusion experiments by Bernadette Straub and their parameter settings.Table 1.

**Experimental design**

The first column perfusion system, established by Straub, transported the lactose solution from a glass bottle into the column using a pressure system that operated at 0.2 to 0.4 bar. The efflux speed was adjusted by a stopcock integrated in the column. The heatable column was tempered via a thermostat (Thermomix ME 852 112/3, B.Braun Melsungen AG, Melsungen, Germany), pumping heated water through the column jacket.

Using the system from Straub as basis, a peristaltic pump (Reglo, Ismatec® – Cole-Parmer GmbH, Wertheim, Germany) was attached to the end of the column, to adjust the efflux speed more precisely (Figure 1). A cycle system was established, transporting the efflux back into the initial glass bottle, to enable repeated column perfusion (Figure 2).

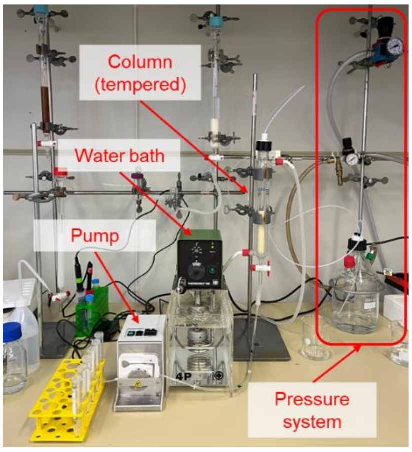

*Figure 1: Set-up of the singe run system.*

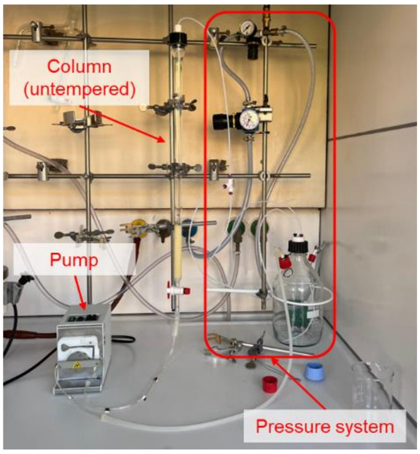

*Figure 2: Set-up of the cycle run system.*

## Perfusion experiments

In initial perfusion experiments conducted by Straub, a lactose solution (4.7 g/l) was passed through the column under varying conditions. Four experiments were performed in which temperature, flow rate, and Lactase loading were varied, and the same lactose solution was collected and perfused repeatedly. The exact perfusion conditions can be seen in Table 1.

*Table 1: Perfusion experiments by Bernadette Straub and their parameter settings.*

| Experiment | Temperature | Perfusion speed | Utilized immobilized Lactase | Duration | Sampeling frequency |
|---|---|---|---|---|---|
| Temperature | 55 °C | 0.375 – 0.9 ml/min | Approach Straub: Column | 30 min | 10 min |
| Repeated perfusion | 37 °C | 0.27 – 1.129 ml/min | Approach Straub: Column | 3x 30 min | 30 min |
| Various flow rates | 37 °C | 0.312 – 1.282 ml/min | Approach Straub: Column | 2h | 10 min |
| Lactase loading | 37 °C | 0.2 - 0.875 ml/min | Approach Straub: Column + R4, R6, R12 + R13 | 4x 10 min | 10 min |

Similar experiments were conducted using the pump controlled system set-up. All experiments were conducted at 22 °C and 37 °C. If not further specified, a basic pump speed of 0.5 ml/min was set.

In the experiment examining temperature variation, a single run perfusion was repeated at 22 °C, 37 °C and 55 °C. Samples were collected for 10 minutes over 60 minutes. Lactase from all reactors of Approach 1 and 2 were used to conduct the perfusion.

The Perfusion speed was increased in another experiment from 0.5 ml/min to 4.5 ml/min and lastly to 9 ml/min. All three perfusions were conducted using the cycle system with a 100 ml lactose solution. During the perfusion at 37 °C, one sample was taken every hour for a total of 6 hours, whereas at 22 °C, samples were collected only in triplicate after 6 hours. Again, all reactors of Approach 1 and 2 were selected for perfusion.

The increased Lactase amount experiment was conducted by adding reactor after reactor into the, at the beginning empty, column. For each added reactor, a single run perfusion was conducted for 30 minutes, collecting three samples in total over 10 minutes. The perfusion at 22 °C used the Lactase from reactor 5, 6, 7, 8, 9 and 10 of Approach 4 in the respective order, while the one at 37 °C was conducted using reactor 1, 2, 5, 6, 7 and 8 of Approach 3, again in the respective order.

Lastly, a long time cycle perfusion experiment was carried out for 72 hours, using the same Lactase as in the Lactase increase experiment and was done for both temperatures respectively. 100 ml lactose was initially added to the cycle system and approximately 0.5 ml was taken for each sample, drawn each day at 7:30, 13:30 and 19:30 o'clock.

All samples were analysed using the Enzytec™ Liquid D-Glucose Test-Kits and a UV-VIS Spectrometer (UVmini-1240, Shimadzu Corporation, Kyoto, Japan), following the manufacturer's instructions, with the exception, that all volumes were reduced by half. Lactose conversion was evaluated based on the glucose yield, defined as the amount of glucose produced relative to its maximum theoretical amount (Eq. I).

$$\text{Glucose yield (\%)} = \frac{c_{\text{max.,actual}}}{c_{\text{max.,theoretical}}} * 100\ \% \qquad \text{I}$$

# 3 RESULTS

The following passages show the results of the initial perfusion experiments performed by Staub and the following experiments using advanced set ups.

## 3.1 Initial column perfusion experiments by Straub performed under variable conditions

The results from the initial perfusion experiments without a pump, performed by Straub, are seen in Table 2 to Table 5.

The results of the experiment with the perfusions at different speeds is shown in Table 2. The perfusion over 125 minutes showed a speed range from 0.025 ml/min up to 1.650 ml/min. At the lowest speed, a yield of 42 % was achieved. The highest yield of 52 % was obtained at speed 0.35 ml/min, while another sample at the same speed only achieved 37 %. At the highest speed, the lowest glucose yield of 13 % was reached. In the perfusion over 60 minutes, a speed range from 0.275 ml/min to 0.675 ml/min was recorded. The lowest perfusion speed showed also the lowest yield with 22 %, while the highest speed showed a yield of 29 %. The highest

yield was reached at the second lowest speed of 0.3 ml/min. Throughout both perfusions, the same yield was achieved by samples with different perfusion speeds.

*Table 2: Glucose yields, and Glucose concentration obtained at different perfusion speeds in two approaches.*

| **Fraction** | **Perfusion speed (ml/min)** | **Glucose (g/l)** | **Glucose yield (%)** | **Fraction** | **Perfusion speed (ml/min)** | **Glucose (g/l)** | **Glucose yield (%)** |
|---|---|---|---|---|---|---|---|
| **Perfusion 1a: Different perfusion speeds, 37 °C** | | | | **Perfusion 1b: Different perfusion speeds, 37 °C** | | | |
| A | 1.05 | 0.451 | 18 | a | 0.275 | 0.532 | 22 |
| B | 0.9 | 0.652 | 26 | b | 0.3 | 0.807 | 33 |
| C | 0.925 | 0.565 | 23 | c | 0.6 | 0.766 | 31 |
| D | 1.375 | 0.413 | 17 | d | 0.525 | 0.714 | 29 |
| E | 1.65 | 0.312 | 13 | e | 0.575 | 0.711 | 29 |
| F | 1.1 | 0.425 | 17 | f | 0.675 | 0.72 | 29 |
| G | 1.5 | 0.388 | 16 | | | | |
| H | 0.675 | 0.519 | 21 | | | | |
| I | 0.5 | 0.624 | 25 | | | | |
| J | 0.35 | 0.926 | 37 | | | | |
| K | 0.35 | 1.282 | 52 | | | | |
| L | 0.025 | 1.048 | 42 | | | | |

Repeating the perfusion of the same Lactase solution three times as displayed in Table 3, showed a severe yield increase. The perfusion with 29 ml showed an yield increase from 21 % over 39 % to 46 %. It presents a much higher increase of 18 % in the first repetition at a lower perfusion speed of 0.675 ml/min when compared to the second repetition at 0.833 ml/min increasing by 7 %. The second approach with a 15 ml Lactase solution showed overall lower yields, increasing from 11 % up to 18 % and finally 26 %. This time, the second perfusion, at a speed of 1.3 ml/min, showed slightly higher increase compared to the first repetition at a speed of 2.6 ml/min.

*Table 3: Glucose concentration and yield with corresponding perfusion speeds for the same lactose solution, after repeated perfusions.*

| Fraction | Perfusion speed (ml/min) | Glucose (g/l) | Glucose yield (%) | Fraction | Perfusion speed (ml/min) | Glucose (g/l) | Glucose yield (%) |
|---|---|---|---|---|---|---|---|
| **Perfusion 2a: Repeated Perfusion of the same Lactase solution (29 ml), 37 °C** | | | | **Perfusion 2b: Repeated Perfusion of the same Lactase solution (15 ml), 37 °C** | | | |
| 1.1 | 0.967 | 0.509 | 21 | 1.2 | 1.875 | 0.27 | 11 |
| 2.1 | 0.675 | 0.958 | 39 | 2.2 | 2.8 | 0.435 | 18 |
| 3.1 | 0.833 | 1.129 | 46 | 3.2 | 1.3 | 0.648 | 26 |

The perfusion experiment at 55 °C (Table 4) showed glucose yields between 26 % and 38 % with an average of approximately 30 % and standard deviation of 6.7 %. In comparison to values with a similar perfusion speed at 37 °C, only little or no increase was observed.

Increasing the amount of immobilized Lactase to 150 % (Table 5) showed an average yield of approximately 29 % with a standard deviation of 10.8 %. Both increase and decrease could be observed, when compared to samples with similar speeds, though certain variations of the speed between the approaches existed.

*Table 4: Glucose concentration and yield with corresponding perfusion speeds from a perfusion at 55 °C.*

| Fraction | Perfusion speed (ml/min) | Glucose (g/l) | Glucose yield (%) |
|---|---|---|---|
| **Perfusion 3: Perfusion at a higher temperature, 55 °C** | | | |
| one | 0.9 | 0.648 | 26 |
| two | 0.425 | 0.662 | 27 |
| three | 0.375 | 0.952 | 38 |

*Table 5: Glucose concentration and yield with corresponding perfusion speeds for the perfusion containing higher amount of immobilized Lactase.*

| Fraction | Perfusion speed (ml/min) | Glucose (g/l) | Glucose yield (%) |
|---|---|---|---|
| **Perfusion 4: Increased amount of immobilized Lactase (150 %), 37 °C** | | | |
| Blank | | | |
| I | 0.875 | 0.381 | 15 |
| II | 0.2 | 0.67 | 27 |
| III | 0.15 | 1.02 | 41 |
| IV | 0.7 | 0.776 | 31 |

## 3.2 Column perfusion at 22 °C, 37 °C and 55 °C

The glucose yield, obtained from perfusions at three different temperatures, is presented in Figure 3. At 22 °C, the lowest yield of 3 % was recorded. Raising the temperature to 37 °C resulted in a 25 % yield. The highest yield of 31.8 % was achieved at 55 °C, showing an approximately tenfold increase compared to the yield at 22 °C.

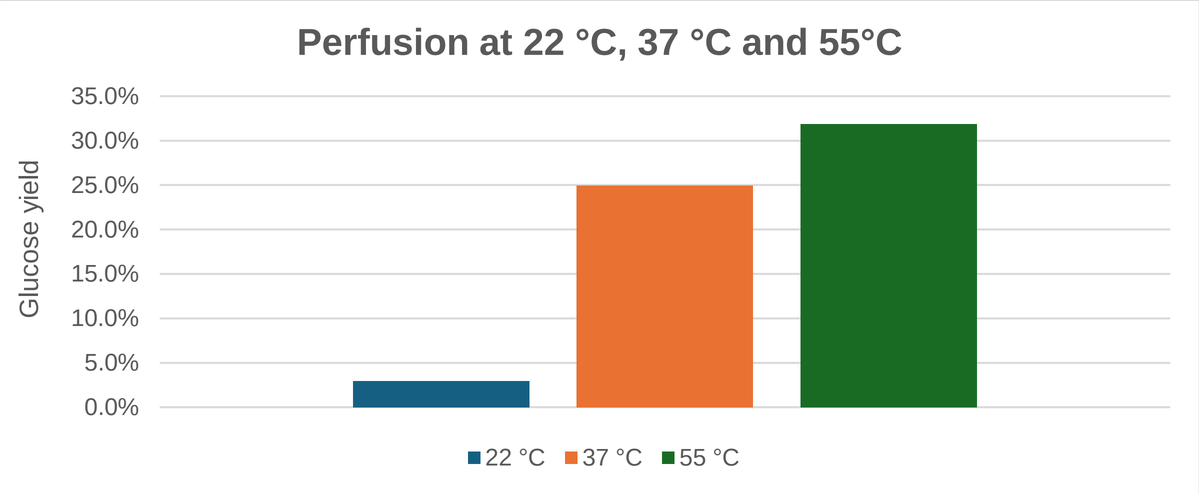


*Figure 3: Glucose yields of different column perfusions at 22 °C, 37°C and 55 °C.*

## 3.3 Perfusion cycle with different pump speeds for 6 hours at 22 °C and 37 °C

The results obtained from the cycle run perfusions after six hours with three different pump speeds at 22 °C as well as 37 °C are shown in Figure 4. The highest yields at each temperature were achieved at the lowest pump speed of 0.5 ml/min, with 21.6 % at 22°C and 30.1 % at 37 °C. At both temperatures, similar yields were obtained at pump speeds of 4.5 ml/min and 9 ml/min. The 22 °C perfusion resulted in a slightly higher yield of 17.9 % at 9 ml/min compared to 17.3 % at 4.5 ml/min. At 37 °C, a slightly lower yield of 25.3 % was observed at a speed of

9 ml/min compared to a 25.9 % yield at 4.5 ml/min. Across all pump speeds, the yields at 22 °C were approximately 8 % lower than the ones at 37 °C.

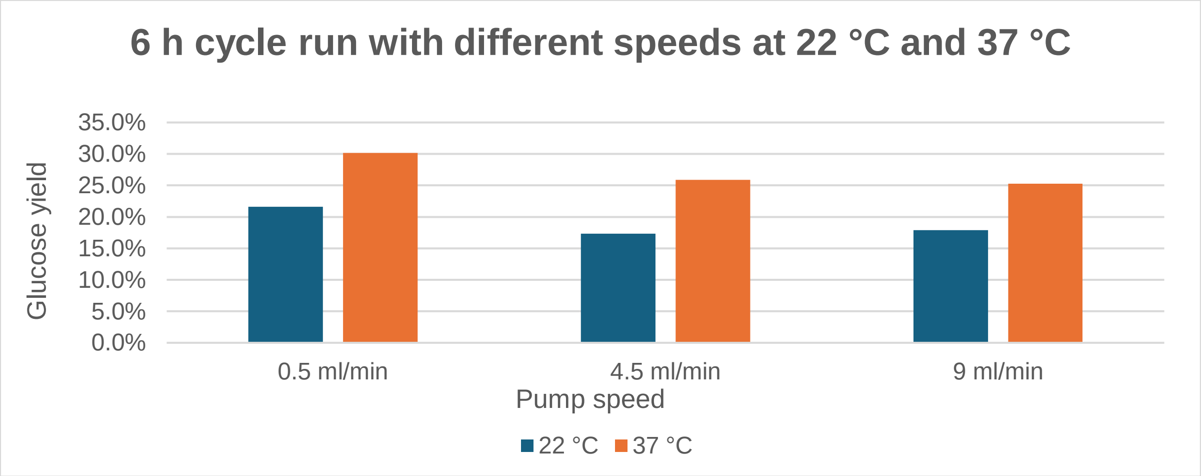


*Figure 4: Glucose yield of cycle run perfusions at 22 °C and 37 °C with different pump speeds.*

Figure 5 shows the hourly development of the glucose yield for the six-hour perfusion at 37 °C, previously shown in Figure 4. Across all speeds, a linear increase in yield was observed. After one hour, the highest yield of 17.1 % was observed at a speed of 0.5 ml/min. Yields of 4.5 ml/min and 9 ml/min were lower, at 6.0 % and 5.4 %, respectively. Over the time of 6 hours, the yields of speed 4.5 ml/min and 9 ml/min increased at a faster rate than at a speed of 0.5 ml/min, leading the final yields described above.

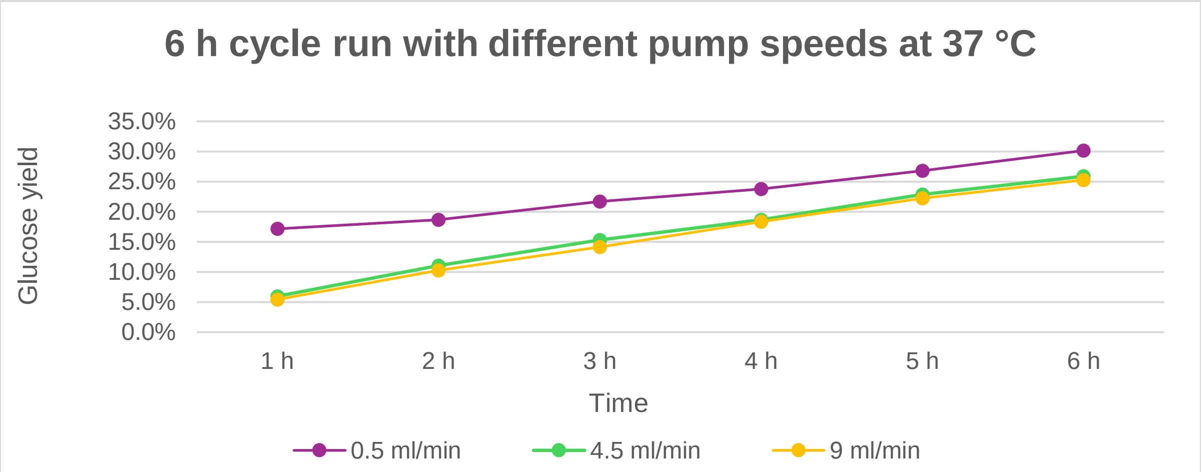


*Figure 5: Hourly development of the glucose yield of three 6 hour cycle run perfusions with different pump speeds at 37 °C.*

## 3.4 Column perfusion with different amounts of immobilized Lactase

Column perfusions with an increasing amount of immobilized Lactase were performed at 22 °C and 37 °C. Figure 6 shows the amount of immobilized Lactase in the column for each perfusion and its corresponding yield. In both perfusions, the yield increased steadily. At 22 °C, the initial yield started at 3.5 % with 2.9 g immobilized Lactase and ended at a total of 17.9 % and 18.1 g immobilized Lactase. At 37 °C, the same initial amount of immobilized Lactase, as at 22 °C, resulted in a glucose yield of 11.1 %, corresponding to an approximately threefold increase. The yield increased to 36.1 % with 17.4 g of Lactase, reaching a higher final value while having a lower enzyme loading, compared to the final yield at 22 °C.

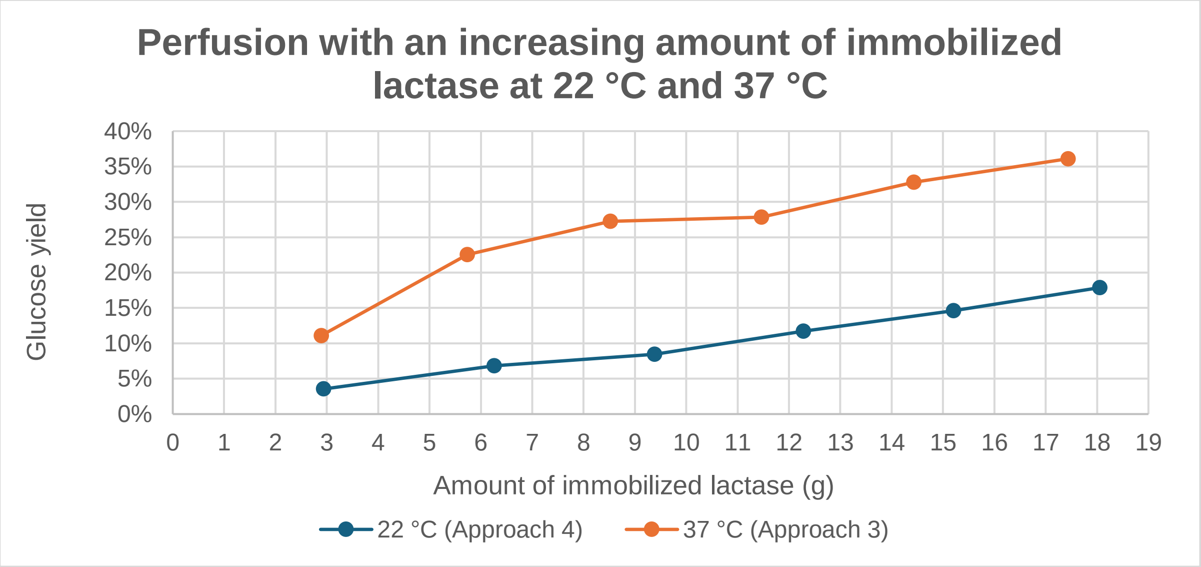


*Figure 6: Perfusion with increased amount of immobilized lactase at 22 °C and 37 °C.*

## 3.5 Perfusion cycle for 72 hours at 22 °C and 37 °C

The results of the 72 hour cycle run perfusions at 22 °C and 37 °C are shown in Figure 7. During the first 6 hours, glucose yields increased rapidly at each temperatures, leading to a yield of 30.9 % and 46.3 % at 22 °C and 37 °C, respectively. At 22 °C, the yield continued to

rise steadily until hour 42, reaching approximately 80 %, after which it stabilized. At 37 °C, 80 % yield was reached by hour 24 and increased further to approximately 90 % at hour 48, after which the yield also stabilized.

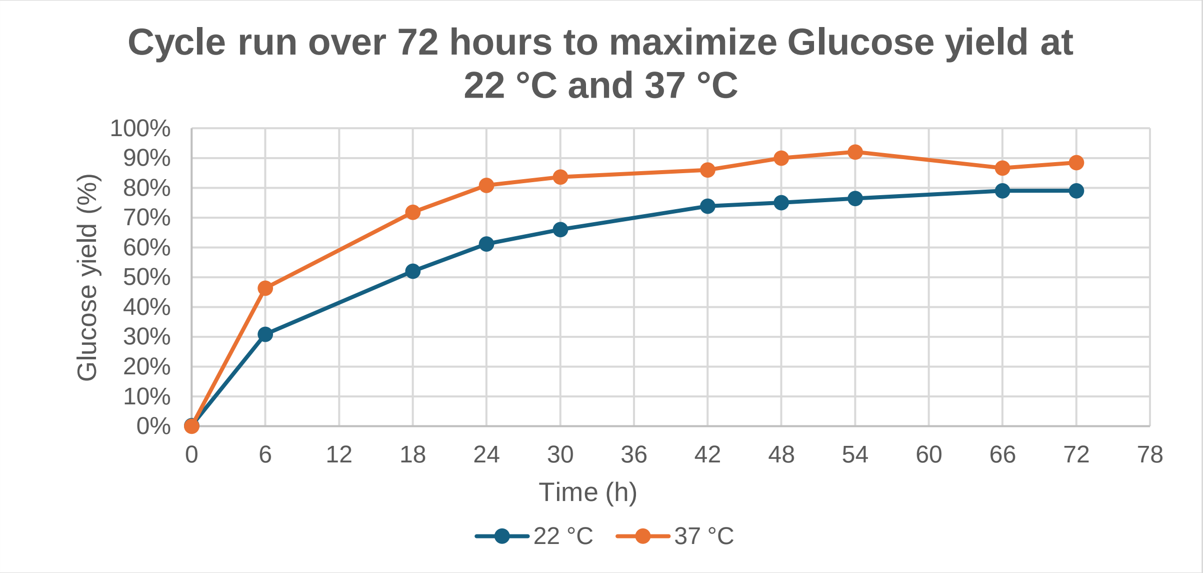


*Figure 7: Glucose yields of cycle run perfusion for 72 hours at 22 °C and 37 °C.*

# 4 DISCUSSION

This study investigated the implementation and optimization of a continuous lactose hydrolysis process using p-phenylenediisothiocyanate-based covalent immobilization of β-D-galactosidase. The efficiency of the immobilization procedure has been characterized in detail previously (11). This data provided the basis for the present implementation of a continuous hydrolysis process. During initial perfusion experiments by Bernadette Straub, flow rates varied continuously due to manual adjustment using the stopcock. While this variability was useful for identifying the effect of the flow rate on the glucose yield, it led to heterogeneous conditions during sampling. This complicates data interpretation, particularly because the results from the glucose test kits were not always unambiguous. The subsequent implementation of a pump controlled flow successfully minimized these fluctuations, leading

to a more consistent perfusion and better comparability within multiple samples. Nevertheless, due to the variety of samples with different speeds, a trend towards higher lactose conversion at lower perfusion speeds could be observed, suggesting a long time of contact to increase lactose conversation. This was also identified in a study from Klein et Al. (12), showing that an increase of the outflow of lactose and whey solution from 2.6 ml/min to 3.4 ml/min resulted in a reduction of the hydrolysis degree in both solutions from over 90 % to 86 % in lactose solution and 80 % in whey solution. Perfusion at the reported temperature optimum of 55 °C for β-Galactosidase, derived from the fungi *Aspergillus oryzae,* showed the highest level of lactose, though at 37 °C, glucose levels were still at a sufficient level (9, 13). Contradicting this, the initial experiment by Straub at 55 °C displayed no enhanced yields compared to the samples at 37 °C with similar speeds. This maybe attributed to the heterogeneous conditions, mentioned above, due to varying perfusion speeds within the samples. In all experiments done additionally at 22 °C, highly lower glucose levels were observed in comparison to the tempered perfusions at 37 °C, signalling insufficient lactose conversion for the use in industrial processes. Overall, this indicates, that temperature plays a crucial role in the optimization of the process. Since microbial contamination is a big problem in β-Galactosidase immobilization, this has to be addressed further (9). Recent literature suggests, that milk hydrolysis should be performed at low temperatures, to reduce microbial growth, contradicting the use of the *Aspergillus oryzae* β-Galactosidase (14, 9). However, another approach to reduce microbial contamination suggested by recent studies is the use of thermostable β-Galactosidases that endure the pasteurization temperature of 65 °C (15, 16). A Study by Farag et. Al. showed, that *Aspergillus oryzae* β-Galactosidase maintains over 50 % of its enzyme activity at 70 °C, indicating the possible use of the enzyme at pasteurization temperatures, to reduce the risk of

a microbial contamination (17). Another aspect of operating at high temperatures is the need for a high thermal stability. Fortunately, Lactase of *Aspergillus oryzae* is known for its high thermostability, that is improved by immobilization (18, 19).

The initial study of an increasing amount of immobilized Lactase by Straub showed no clear increase in glucose yields, though a comparison is complicated due to the absence of similar conditions. An improved experimental set-up using the pump controlled system, presented a linear correlation between the lactose amount and glucose yield. This suggests, that the amount of immobilized Lactase can be further increased to lead to higher glucose yields. Though, due to the limited availability of immobilized Lactase and its production costs, the increase in amount of immobilized Lactase is restricted. Furthermore, an increase in yield is highly affected by the immobilized enzyme efficiency.

Since single run perfusions showed yields far from 100 %, a circular perfusion set-up was implemented, mimicking a possible industrial process at a small scale. The repeated perfusions of the same lactose solution by Straub showed a clear increase in yield, suggesting that the more cycles the Lactase undergoes, the higher the yield should be, while the time of contact also needs to be sufficient. Hence, a compromise between the number of cycles and time of contact hat to be examined by variation of the perfusion speed. Results after 6 hours showed the highest lactose conversion in the perfusion with the slowest perfusion speed, indicating the higher importance of contact time over numbers of cycles. Contradicting this, the development of the glucose yield over the 6 hours displays a faster increase at higher speeds, suggesting that the duration plays a crucial role in determining the optimal perfusion speed.

Long term cycle perfusion, at optimal conditions showed a stagnation in lactose conversion at 90 % after 48 hours, although 80 % was already achieved after 24 hours. The same trend at lower levels has been observed in the 22 °C perfusion, supporting the validity of the curve. The stagnation below a 100 % yield might be addressed to the inhibitory effect of the hydrolysis product galactose on β-Galactosidase (9). This limitation can also be observed in another immobilization approach using the entrapment method, achieving only 87 % of hydrolysis degree after 36 hours (14). Another immobilization approach, using alginate immobilized *Aspergillus oryzae* β-Galactosidase, also shows limited degree of hydrolysis at 70.9 % but was only carried out for 12 hours and as a batch process (20). At this point it needs to be highlighted, that the mentioned approaches were carried out using skim milk, while the experiments of this study used a lactose solution containing a 10 times lower concentration than milk. Therefore, qualitative comprehension of the approaches is impossible. Nevertheless, this highlights the fact, that further improvement in yield is inevitable for industrial processes. The optimal perfusion speed could be examined at longer cycle perfusion durations for optimal balance of cycle number and contact time. Furthermore, the reduction of the possibility for microbial contamination should be addressed by testing the effectivity of the immobilized ß-Galactosidase at pasteurization temperatures, or investigating the use of low temperature operating ß-Galactosidase. However, a first approach of a continuous hydrolysis process using immobilized Lactase in a fixed bed reactor was successfully established.

**Supporting Information.**

The following files are available free of charge. Raw data perfusion experiments Bernadette Straub (XLSX) Raw data perfusion experiments Tabea Boehme including long term cycle perfusion experiments (XLSX)

**Author Contributions**

Tabea Boehme: Writing - Original Draft, Data Curation, Investigation. Bernadette Straub: Data Curation, Investigation. Ursula Eschenhagen: Resources, Methodology. Magnus S. Schmidt: Conceptualization, Supervision, Writing – review & editing.

No funds, grants, or other support was received.

**Acknowledgement**

We would like to thank the ReAching program of the faculty Health, Medical and Life Sciences, Furtwangen University, for support.

***Author information***

*Tabea L. Boehme, EMail: boehmetabea@gmail.com*

*Bernadette Straub: EMail: bernadette@straub.id*

*Ursula Eschenhagen, Email: ursula.eschenhagen@hs-furtwangen.de*

*Magnus S. Schmidt, Email: magnus.schmidt@hs-furtwangen.de*

***Credit***

*Photograph courtesy of Tabea L. Boehme. Copyright 2025 Tabea L. Boehme. Images are free domain.*

## 5 References

1. Kalathinathan P, Sain A, Pulicherla K, Kodiveri Muthukaliannan G. A Review on the Various Sources of β-Galactosidase and Its Lactose Hydrolysis Property. Curr Microbiol 2023; 80(4):122.

2. Matthews BW. The structure of E. coli beta-galactosidase. C R Biol 2005; 328(6):549–56.

3. Dekker PJT, Koenders D, Bruins MJ. Lactose-Free Dairy Products: Market Developments, Production, Nutrition and Health Benefits. Nutrients 2019; 11(3).

4. Bernal C, Urrutia P, Illanes A, Wilson L. Hierarchical meso-macroporous silica grafted with glyoxyl groups: opportunities for covalent immobilization of enzymes. N Biotechnol 2013; 30(5):500–6.

5. Rizzo PV, Harwood WS, Drake MA. Consumer desires and perceptions of lactose-free milk. J Dairy Sci 2020; 103(8):6950–66.

6. Veum L, Hanefeld U. Carrier enabled catalytic reaction cascades. Chem Commun (Camb) 2006; (8):825–31.

7. Damin BIS, Kovalski FC, Fischer J, Piccin JS, Dettmer A. Challenges and perspectives of the β-galactosidase enzyme. Appl Microbiol Biotechnol 2021; 105(13):5281–98.

8. Dayi DI, Eschenhagen U, Seidinger H, Schneider H, Schmidt MS. p-Phenylene Diisothiocyanate-Based Covalent Immobilization of β-d-Galactosidase and Determination of Enzyme Activity by Cleavage of X-Gal and ONPG on Solid Support. ACS Omega 2023; 8(30):27585–96.

9. Dekker P. Enzymes Exogenous to Milk in Dairy Technology: β-d-Galactosidase. In: Reference Module in Food Science. Elsevier; 2019.

10. sanotact GmbH. Sanotact lactase 24 000 6h depot (40 lactase tablets) Lactose tablets with depot effect for lactose intolerance & milk intolerance immediate effect & 6h long-term depot 24 000 depot 40 tablets [cited 2025 Aug 27]. Available from: URL: https://www.gosupps.com/sanotact-lactase-24-000-6h-depot-40-lactase-tablets-lactose-tablets-with-depot-effect-for-lactose-intolerance-milk-intolerance-immediate-effect-6h-long-term-depot-24-000-depot-40-tablets.html?

11. Boehme T, Straub B, Eschenhagen U, Schmidt M. Dataset on the analysis of β-galactosidase immobilization efficiency on AMP resin in syringe and column reactors; 2025.

12. Klein MP, Fallavena LP, Da Schöffer JN, Ayub MAZ, Rodrigues RC, Ninow JL et al. High stability of immobilized β-D-galactosidase for lactose hydrolysis and galactooligosaccharides synthesis. Carbohydr Polym 2013; 95(1):465–70.

13. Zolnere K, Ciprovica I. The comparison of commercially available β-galactosidases for dairy industry : review. In: Latvia University of Agriculture; 2017. p. 215–22 (Research for rural development).

14. Schulz P, Rizvi SS. Hydrolysis of Lactose in Milk: Current Status and Future Products. Food Reviews International 2023; 39(5):2875–94.

15. Ionata E, Marcolongo L, La Cara F, Cetrangolo GP, Febbraio F. Improvement of functional properties of a thermostable β-glycosidase for milk lactose hydrolysis. Biopolymers 2018; 109(10):e23118.

16. Marín-Navarro J, Talens-Perales D, Oude-Vrielink A, Cañada FJ, Polaina J. Immobilization of thermostable β-galactosidase on epoxy support and its use for lactose hydrolysis and galactooligosaccharides biosynthesis. World J Microbiol Biotechnol 2014; 30(3):989–98.

17. Farag AM, Hassan MA. Purification, characterization and immobilization of a keratinase from Aspergillus oryzae. Enzyme and Microbial Technology 2004; 34(2):85–93.

18. Hirohara H, Yamamoto H, Kawano E, Nagase T. Continuous Hydrolysis of Lactose in Skim Milk and Acid Whey by Immobilized Lactase of Aspergillus Oryzae. In: Chibata I, Fukui S, Wingard LB, editors. Enzyme Engineering. Boston, MA: Springer US; 1982. p. 295–7.

19. Bayramoglu G, Cimen AG, Arica MY. Immobilisation of β-galactosidase onto double layered hydrophilic polymer coated magnetic nanoparticles: Preparation, characterisation and lactose hydrolysis. International Dairy Journal 2023; 138:105545.

20. Katrolia P, Liu X, Li G, Kopparapu NK. Enhanced Properties and Lactose Hydrolysis Efficiencies of Food-Grade β-Galactosidases Immobilized on Various Supports: a Comparative Approach. Appl Biochem Biotechnol 2019; 188(2):410–23.

**ABSTRACT:** In this study, a continuous lactose hydrolysis process in a fixed-bed reactor was developed using β-galactosidase covalently immobilized on resin beads via 1,4-phenylenediisothiocyanate (PDC) as linker. Process conditions, including temperature, enzyme loading, perfusion speed, and repeated perfusion of the same substrate solution were systematically varied. The highest glucose yields were obtained at 55 °C, with increased yields observed at low perfusion speeds, high enzyme loadings, and upon repeated perfusions. Under optimized cycle perfusion over 72 h, final lactose conversion reached approximately 90 % at 37 °C and 80 % at 22 °C. A hydrolysis process in a fixed-bed reactor was successfully established, although further optimization is required.

Click here to access/download
**Supplementary File**
Raw data perfusions_Bernadette Straub.xlsx

Click here to access/download
**Supplementary File**
Raw data perfusions_Tabea Boehme.xlsx

Click here to access/download
**Co-submission to Data in Brief**
Data in Brief_manuscript_final.docx

**Declaration of interests**

☒The authors declare that they have no known competing financial interests or personal relationships that could have appeared to influence the work reported in this paper.

☐The authors declare the following financial interests/personal relationships which may be considered as potential competing interests:
